# Biomod2 Modeling for Predicting Suitable Distribution of Bamboo Bat (*Tylonycteris pachypus*) Under Climate Change

**DOI:** 10.3390/ani15081164

**Published:** 2025-04-17

**Authors:** Kai Chen, Weiwei Shao, Yalei Li, Lijin Wang, Zhihua Lin, Ling Guo, Li Wei

**Affiliations:** College of Ecology, Lishui University, Lishui 323000, China; lsxyckk@163.com (K.C.); shaoweiwei2005@163.com (W.S.); liyalei@lsu.edu.cn (Y.L.); lsxywlj@126.com (L.W.); zhlin1015@126.com (Z.L.)

**Keywords:** climate change, environmental factor, distribution pattern, *Tylonycteris pachypus*, Biomod2

## Abstract

Climate change significantly impacts species distribution and survival, particularly for habitat specialists with limited dispersal abilities. This study investigates the current and future distribution of *Tylonycteris pachypus*, one of the world’s smallest bats specialized in bamboo-dwelling, using ensemble modeling approaches. Based on comprehensive occurrence data and seven environmental variables, we developed an ensemble model using the Biomod2 platform, achieving high predictive accuracy (AUC: 0.981, TSS: 0.877). Three environmental variables were identified as crucial determinants: minimum temperature of the coldest month (40.90% contribution), maximum temperature of the warmest month (38.38%), and precipitation of the wettest quarter (11.09%). Currently, highly suitable habitats (291.893 × 10^4^ km^2^) are concentrated in three main regions: southern China and Indochina Peninsula, Myanmar–Bangladesh–northeastern India, and isolated areas in southwest India and Thailand. Under future climate scenarios, particularly SSP585, suitable habitats are projected to decrease substantially (64.4% reduction by 2090s), with a notable northward shift in distribution. However, the species’ limited dispersal ability, specific habitat requirements, and geographical barriers may constrain its capacity to track these climate-driven changes. Our findings highlight the vulnerability of *T. pachypus* to climate change and emphasize the need for targeted conservation strategies, including protecting climate-resilient habitats and maintaining bamboo forest corridors. This study provides a comprehensive framework for monitoring and conserving this specialized species under climate change, while considering its unique ecological constraints and dispersal limitations.

## Simple Summary

Climate change remains a critical focus in ecological research. This study uses an ensemble modeling approach to predict potential suitable habitats for the bamboo bat (*Tylonycteris pachypus*, Temminck, 1840), based on current geographic occurrence data and key environmental variables. Our optimized ensemble model outperformed individual models in simulation accuracy. We identified three environmental factors that significantly influence *T. pachypus*’s distribution. Currently, the suitable habitat for *T. pachypus* mainly consists of three regions, characterized by a warm, humid tropical monsoon climate. Looking ahead, our models project a substantial decrease in suitable habitats for *T. pachypus* under various climate change scenarios. This research not only enhances our understanding of *T. pachypus*’s population ecology but also provides valuable insights for its conservation. Furthermore, our approach and findings may be applicable to the study and protection of other bat species facing similar climate-related challenges.

## 1. Introduction

Climate change has emerged as one of the most pressing global challenges, with far-reaching implications for ecosystems worldwide [1]. Recent evidence from the Sixth Assessment Report (AR6) of the Intergovernmental Panel on Climate Change (IPCC) reveals an alarming increase in global surface temperatures by 1.09 °C (0.95 to 1.20 °C) from 1850–1900 to 2011–2020 [2]. This accelerating warming trend, coupled with increasing frequency of extreme weather events [3,4], is fundamentally altering species’ geographical distributions and survival patterns [5,6,7]. As climate conditions shift, both flora and fauna experience significant range shifts, with projections indicating that approximately 51% of plant species could see their geographical ranges reduced by half by 2100 [8]. Unless deep reductions in CO_2_ and other greenhouse gas emissions occur in the coming decades, global warming of 1.5 °C and 2 °C will be exceeded during the 21st century [2], potentially accelerating species extinction rates and modifying plant phenology and growing seasons [9,10]. Among the various impacts, the alteration of habitat suitability emerges as one of the most critical factors jeopardizing species survival [11], making it imperative to investigate species’ responses to climate change for maintaining local ecological equilibrium and preserving biodiversity [9,12].

To better understand and predict species’ responses to climate change, Species Distribution Models (SDMs) have become essential tools in ecological research [13,14,15]. These models effectively link species’ ecological niches with environmental factors to forecast potential distribution patterns under various climatic scenarios [5,16,17]. Among various modeling approaches, ensemble modeling has emerged as a particularly robust method due to its ability to integrate multiple algorithms and reduce individual model uncertainties [18,19]. The ensemble model (EM) implemented in the Biomod2 platform has demonstrated exceptional accuracy in predicting species distributions and identifying key environmental drivers [5], as evidenced by successful applications across diverse taxa such as *Populus davidiana* [20], *Ochotona curzoniae* [21], and *Boselaphus tragocamelus* [10].

*Tylonycteris pachypus* (Temminck, 1840), one of the world’s smallest mammals, represents a unique case of habitat specialization among vespertilionid bats [22]. This species is distinctively adapted to inhabit narrow bamboo tubes in tropical and subtropical humid monsoon climates, possessing specialized morphological features including a remarkably flat skull and unique adhesive pads [23]. While previous research has explored various aspects of *T. pachypus*, including its echolocation capabilities [24], social behavior [25,26], feeding ecology [27], genetic structure [28], and mitochondrial genomics [29], a critical knowledge gap remains regarding its vulnerability to climate change. The species’ highly specialized habitat requirements, coupled with ongoing habitat fragmentation due to rapid urbanization [30], make it particularly susceptible to environmental changes. Furthermore, as a species dependent on specific bamboo forest ecosystems, *T. pachypus* could serve as an important indicator of climate change impacts on specialized forest-dwelling species. Given these considerations, we conducted a comprehensive assessment of *T. pachypus*’s distribution under four climate scenarios (SSP126, SSP245, SSP370, and SSP585) for both current conditions and future projections (2041–2060 and 2081–2100), aiming to (1) predict current and future potential distributions using ensemble modeling in Biomod2, (2) identify key environmental constraints on its distribution, and (3) evaluate dynamic changes in climate-appropriate areas under future scenarios. This research not only addresses a significant gap in our understanding of this unique species but also provides valuable insights for developing effective conservation strategies in the face of global environmental change.

## 2. Materials and Methods

### 2.1. Data Collection

**Figure 1:**
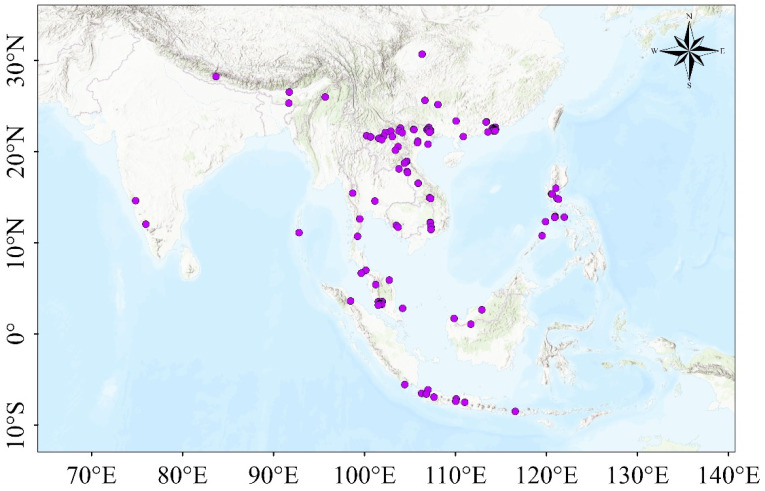
Geographical distribution of *Tylonycteris pachypus* occurrence points.

To establish a comprehensive dataset of *T. pachypus* distribution, we compiled occurrence records from multiple sources, including field surveys, published literature, and the Global Biodiversity Information Facility (GBIF) database. The spatial distribution of these records was carefully mapped and analyzed using ArcGIS Pro (Environmental Systems Research Institute, Inc., California, USA, Reference ID: 602162530176). Data quality control was performed following the method described by Dhami et al. (2023) [9], with adaptations to account for the specific dispersal characteristics of *T. pachypus*. This process involved several steps: (i) Removal of duplicate records to prevent overrepresentation of certain locations. (ii) Elimination of occurrence points that fell outside the species’ known distribution range, based on expert knowledge and published literature. (iii) Spatial filtering to reduce sampling bias. We employed a grid-based approach, where a single occurrence point was randomly selected from each 5 km × 5 km grid cell if multiple records were present. This step was implemented using the Euclidean Distance package in ArcGIS. Following this rigorous data cleaning and filtering process, we retained a total of 109 unique distribution points for *T. pachypus* (Figure 1). This refined dataset forms the foundation for our subsequent species distribution modeling efforts.

### 2.2. Environmental Variables Selection and Processing

To comprehensively assess the factors influencing the distribution of *T. pachypus*, we incorporated a diverse set of environmental variables into our potential distribution model. These variables encompassed bioclimatic (19 variables), topographic (elevation), and anthropogenic (land use and cover change) factors (Table 1). Current climate data were sourced from the WorldClim database (http://www.worldclim.org/; accessed on 20 June 2023) at a spatial resolution of 2.5 arc-minutes (approximately 5 km × 5 km). This dataset includes 19 bioclimatic variables representing temperature and precipitation patterns from 1970 to 2000 [31]. For future climate projections, we utilized data from the BCC-CSM2-MR climate model, part of the Coupled Model Intercomparison Project Phase 6 (CMIP6) [8,32]. We focused on two time periods: the 2050s (2041–2060) and 2090s (2081–2100), under four contrasting emissions scenarios: SSP126 (low emissions), SSP245 (intermediate emissions), SSP370 (high emissions), and SSP585 (very high emissions) [33]. Topographic data (elevation) were derived from the GEBCO digital elevation model (http://www.gebco.net; accessed on 6 April 2023) at 5 km resolution. Anthropogenic influence was represented by land use and cover change data, obtained from the 2020 global land cover database (https://maps.elie.ucl.ac.be/CCI/viewer/index.php; accessed on 6 October 2024).

To mitigate potential overfitting and address multi-collinearity among environmental variables, we employed a two-step variable selection process [34]: (i) Principal Component Analysis (PCA) was conducted using the FactoMineR package in R v 4.3.1 to identify the main axes of variation in the environmental data; (ii) Pairwise Pearson’s correlations were calculated among all variables (Supplementary Figure S1). Based on the combined results of the PCA and correlation analysis, we excluded variables with low percentage contribution and high correlation coefficients (|r| ≥ 0.7) to minimize confounding effects [9]. This rigorous selection process resulted in a final set of 7 environmental variables for our ensemble modeling approach: mean diurnal range (Bio2), max temperature of the warmest month (Bio5), min temperature of the coldest month (Bio6), precipitation of the wettest quarter (Bio16), precipitation of the driest quarter (Bio17), precipitation of the warmest quarter (Bio18), and land use and cover change (Lucc) (Table 1).

### 2.3. Model Selection and Technical Framework

The selection of appropriate modeling techniques is crucial for accurate species distribution prediction. Common SDM approaches include Generalized Linear Models (GLMs), Generalized Boosted Models (GBMs), Generalized Additive Models (GAMs), Classification Tree Analysis (CTA), Artificial Neural Networks (ANNs), Rectilinear Envelope models similar to BIOCLIM (SRE), Flexible Discriminant Analysis (FDA), Multivariate Adaptive Regression Splines (MARS), Random Forests (RFs), and Maximum Entropy Models (MaxEnt). Each method has its own principles and algorithms, offering different advantages in species distribution simulation. To overcome the limitations of individual models and enhance prediction accuracy, we adopted an ensemble modeling approach that integrates multiple algorithms to provide more robust predictions.

### 2.4. Model Construction and Evaluation

We employed the Biomod2 package in R version 4.3.1 (http://www.r-project.org/, accessed on 5 June 2023) to model the potential distribution of *T. pachypus* for both current and future climate scenarios. Our approach was based on methods described by Dhami et al. (2023) [9], Zhao et al. (2024) [21], and Zhang et al. (2024) [35] utilizing an ensemble modeling framework.

#### 2.4.1. Model Construction

Initially, we implemented 10 individual models available in Biomod2: Artificial Neural Networks (ANNs), Classification Tree Analysis (CTA), Flexible Discriminant Analysis (FDA), Generalized Boosted Models (GBMs), Generalized Linear Models (GLMs), Multivariate Adaptive Regression Splines (MARS), Maximum Entropy (MaxEnt), Random Forests (RFs), Surface Range Envelope (SRE), and Extreme Gradient Boosting (XGBOOST). We then constructed ensemble models: an optimized ensemble model (EM) comprising three high-performing models: GBM, MARS, and GLM.

#### 2.4.2. Model Training and Validation

The dataset was randomly partitioned into 80% for training and 20% for testing [35,36]. We performed 10 replicates for each model to ensure robustness. One thousand pseudo-absence points were randomly generated globally and replicated once [9,37]. Finally, we select only those with AUC values greater than 0.9 and TSS values greater than 0.7 as the base models for final modeling [35].

#### 2.4.3. Model Evaluation

We employed two widely used metrics to assess model performance: area under the receiver operating characteristic curve (AUC) and true skill statistic (TSS) [21,38,39]. For AUC, a value less than or equal to 0.7 indicates poor model performance, a value between 0.7 and 0.9 indicates moderate performance, and a value above 0.9 indicates excellent performance [40]. For TSS, a value between 0.2 and 0.5 indicates poor model performance, a value between 0.5 and 0.8 indicates moderate performance, and a value above 0.8 indicates excellent performance [41].

#### 2.4.4. Habitat Suitability Classification

The ensemble model predictions were imported into ArcGIS Pro for spatial analysis and visualization. Using the natural breaks (Jenks) method, we classified habitat suitability into four categories: unsuitable habitat (*p* < 0.2), poorly suitable habitat (0.2 ≤ *p* < 0.4), moderately suitable habitat (0.4 ≤ *p* < 0.6), and highly suitable habitat (*p* ≥ 0.6) [42]. This comprehensive modeling and evaluation approach allows for a robust assessment of *T. pachypus*’s potential distribution under current and future climate scenarios.

## 3. Results

### 3.1. Model Performance Evaluation

The performance of individual models and ensemble models was evaluated using two metrics: area under the curve (AUC) and true skill statistic (TSS) (Table 2). The optimized ensemble models (EMs) demonstrated superior performance compared to individual models across all evaluation metrics. Among the 10 individual models, Gradient Boosting Machine (GBM) exhibited the highest average accuracy, achieving an AUC score of 0.963 and TSS score of 0.766. The optimized ensemble model significantly outperformed all individual models, reaching an AUC score of 0.981 and TSS score of 0.877. Moreover, both the standard deviation and coefficient of variation of AUC and TSS in the optimized ensemble model were lower than those of individual models, highlighting the enhanced stability and predictive capability of the ensemble approach.

### 3.2. Key Factor Influencing the Spatial Distribution

Our model identified three critical environmental variables that collectively explained 90.37% of *T. pachypus* distribution patterns (Table 1). The minimum temperature of the coldest month (Bio6) emerged as the strongest predictor, contributing 40.90% to the model’s explanatory power, followed by the maximum temperature of the warmest month (Bio5, 38.38%) and the precipitation of the wettest quarter (Bio16, 11.09%).

**Figure 2:**
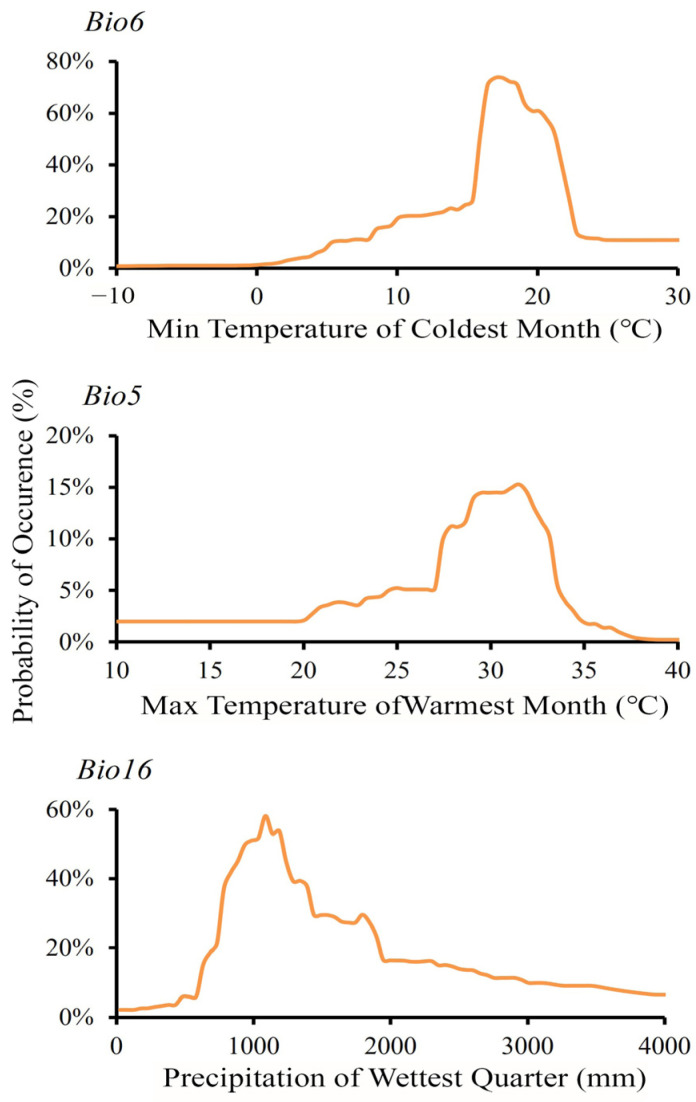
Response curve of dominant environmental factors.

Response curve analysis (Figure 2) revealed distinct environmental preferences of *T. pachypus*: optimal habitat conditions were characterized by minimum temperatures ranging from 15.9 °C to 21.2 °C during the coldest month, maximum temperatures between 27.8 °C and 33.2 °C during the warmest month, and precipitation between 836.08 mm and 1240.12 mm during the wettest quarter.

### 3.3. Potential Suitable Habitats for Current Climate

**Figure 3:**
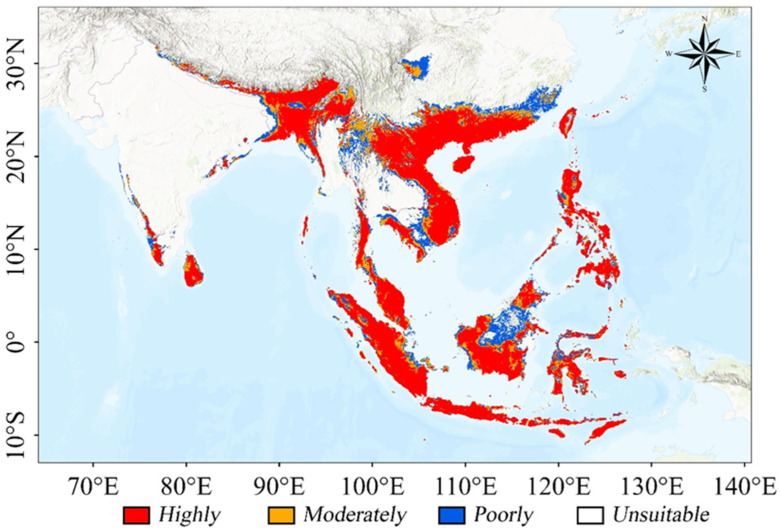
Distribution map of suitable habitat of *Tylonycteris pachypus* under current climate.

The ensemble model predictions revealed complex patterns of *T. pachypus* habitat suitability across Southeast and South Asia under current climate conditions (Figure 3). The total suitable habitat area encompasses approximately 446.505 × 10^4^ km^2^, representing 37.10% of the study area (Table 3). These suitable habitats are primarily distributed across three major regions. The largest and most favorable region extends through southern China (including Taiwan, Guangdong, Hainan, Guangxi, and Guizhou provinces), Vietnam, Laos, Cambodia, Philippines, Malaysia, Singapore, and Indonesia. The second major region comprises Myanmar, Bangladesh, southwestern China (Yunnan and Xizang), northeastern India, Bhutan, and Nepal. A third, smaller but significant region of habitat suitability exists in southwest India, southwest Thailand, and the Sichuan Basin of China. Among these regions, highly suitable habitats cover a total area of 291.893 × 10^4^ km^2^. Throughout its distribution range, *T. pachypus* shows a strong association with warm, humid tropical monsoon climate zones.

### 3.4. Potential Suitable Habitats for Future Scenarios

**Figure 4:**
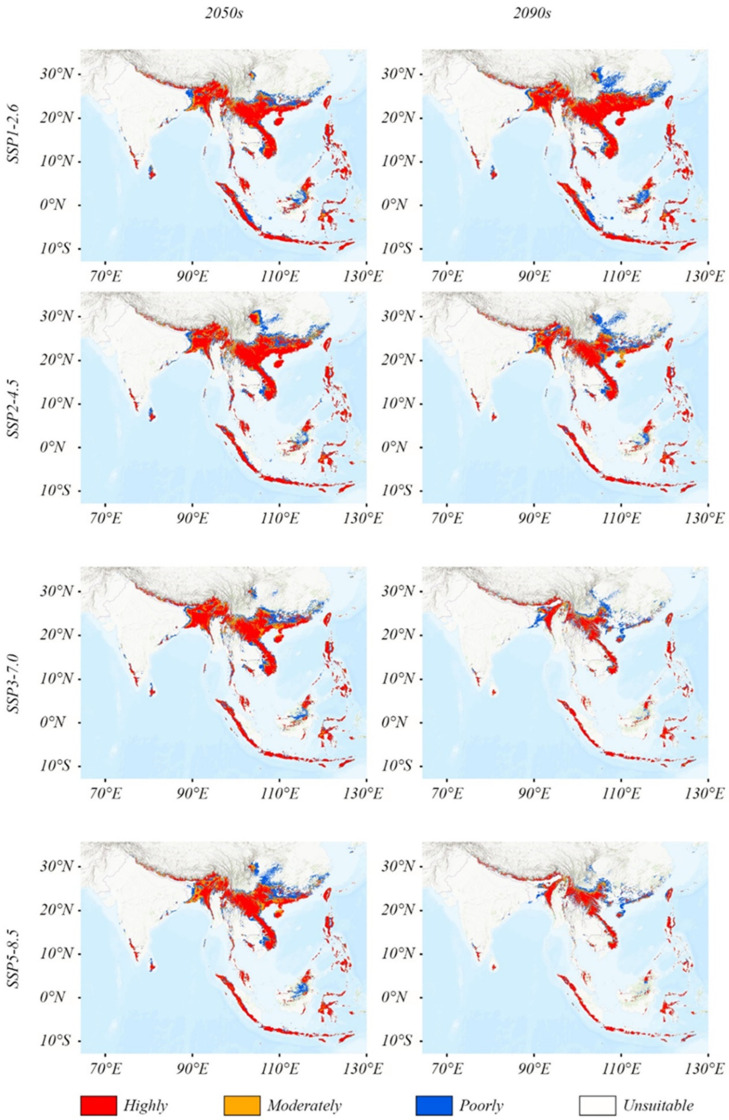
Changes in the distribution pattern of highly and moderately suitable habitats of *Tylonycteris pachypus* under different climatic conditions.

The ensemble model projections revealed substantial changes in *T. pachypus* habitat suitability across different future climate scenarios (Table 3 and Figure 4). Under most scenarios, the suitable habitat area shows a significant reduction by the 2050s and 2090s compared to the present, with the exception of a slight increase under the SSP126 scenario.

The SSP126 scenario predicts the smallest change in habitat suitability, with a 29% reduction (129.517 × 10^4^ km^2^) by the 2050s, followed by a 17.1% increase (54 × 10^4^ km^2^) by the 2090s relative to the 2050s. Under SSP245, the suitable area decreases by 27.3% (122.045 × 10^4^ km^2^) by the 2050s, with a further 15.2% reduction (49.48 × 10^4^ km^2^) by the 2090s. The SSP370 scenario projects a more severe decline, with a 34.5% reduction (154.179 × 10^4^ km^2^) by the 2050s and a 61.2% reduction (273.228 × 10^4^ km^2^) by the 2090s. The most dramatic changes occur under SSP585, where the suitable habitat decreases by 36.1% (161.096 × 10^4^ km^2^) by the 2050s and 64.4% (287.410 × 10^4^ km^2^) by the 2090s. Among all scenarios, SSP585 projects the most substantial habitat loss for both time periods.

## 4. Discussion

### 4.1. Efficacy of Ensemble Modeling and Key Environmental Factors

The application of ensemble modeling techniques in species distribution prediction has gained significant traction due to their superior performance compared to individual models [43,44]. Our study, focusing on *T. pachypus*, one of the world’s smallest mammals, known for its unique roosting behavior in bamboo stem internodes [22,45], successfully demonstrates the advantages of this approach. By employing the ensemble model within the Biomod2 framework, we achieved remarkably high scores of AUC and TSS, surpassing the performance of all individual models. This result aligns with findings from recent studies on diverse species such as *Larix gmelinii* [15], *Ochotona curzoniae* [21], and *Moschus moschiferus* [46], where ensemble models consistently demonstrated higher prediction accuracy compared to single-model approaches.

Our modeling results reveal that the current suitable habitat of *T. pachypus* is predominantly distributed across Southeast and South Asia, from the tropical south to the Tropic of Cancer and northern subtropical areas [23,47]. These regions provide essential ecological conditions for *T. pachypus*, characterized by three key environmental factors: minimum temperature of the coldest month (Bio6, 40.90% contribution), maximum temperature of the warmest month (Bio5, 38.38%), and precipitation of the wettest quarter (Bio16, 11.09%). The dominance of temperature-related variables in our model highlights their crucial role in determining the species’ distribution. Response curves indicate that *T. pachypus* thrives in habitats with minimum temperatures between 15.9 °C and 21.2 °C in the coldest month, maximum temperatures between 27.8 °C and 33.2 °C in the warmest month, and precipitation between 836.08 mm and 1240.12 mm during the wettest quarter. These specific requirements reflect the species’ physiological constraints and ecological adaptations [48].

The strong influence of temperature variables on *T. pachypus* distribution can be explained by both direct and indirect ecological mechanisms. Temperature directly affects bat physiological processes and energy expenditure, while also influencing prey (insect) abundance and activity patterns [26]. *T. pachypus* primarily feeds on small Diptera and Hymenoptera insects [27], whose abundance and distribution are temperature dependent. Additionally, temperature and precipitation patterns significantly influence bamboo forest growth [49], which provides essential roosting sites for *T. pachypus*. Males of this species typically disperse only about 1 km from their natal roosts [50], suggesting that the availability of suitable bamboo habitats within their limited dispersal range is crucial for population persistence.

### 4.2. Current Distribution and Ecological Preferences of T. pachypus

Our ensemble model predictions reveal distinct patterns in the current distribution of *T. pachypus*, with highly suitable habitats concentrated in three main regions. These regions share common characteristics of year-round humid and warm climates, which provide optimal conditions for both *T. pachypus* and its primary habitat, bamboo forests [51].

Interestingly, our model predicts suitable habitats in certain areas where *T. pachypus* has not been recorded, particularly in Taiwan and Hainan Province, China. This discrepancy between predicted and observed distributions can be attributed to several biological and physical constraints. *T. pachypus*’s small body size (average mass 3.3 g) and wing morphology (wing area 5820.9 mm^2^) make it particularly vulnerable to dehydration and limit its dispersal capabilities [52,53]. The species’ large, highly vascularized wing and tail membranes, while advantageous for heat dissipation during flight [54], may paradoxically restrict long-distance movements.

Geographical barriers play a crucial role in limiting *T. pachypus*’s distribution. The Qiongzhou Strait, between Hainan and mainland China, and the Taiwan Strait represent significant barriers to dispersal. While our model estimates the current potential high-suitability habitat area at 291.893 × 10^4^ km^2^ (24.3% of the study area), this figure likely overestimates the actually accessible habitat. Ongoing urbanization has led to significant fragmentation and loss of bamboo forests around villages [26], creating additional barriers to dispersal and reducing habitat connectivity. This combination of physiological constraints, geographical barriers, and habitat fragmentation helps explain why *T. pachypus* may be absent from areas that appear climatically suitable.

### 4.3. Multiple Constraints on Distribution and Dispersal

The distribution pattern of *T. pachypus* is shaped by multiple interacting constraints, including physiological limitations, behavioral traits, and landscape features. While climate suitability provides the fundamental framework for potential distribution, the realized distribution is further filtered through these additional constraints. The species’ specialized adaptations for bamboo-dwelling, including its flat skull and small body size [22], while advantageous for its unique niche, paradoxically increase its vulnerability to environmental changes and limit its colonization abilities.

Energy constraints play a crucial role in limiting *T. pachypus*’s distribution and dispersal. The species’ small body size results in a high surface-to-mass ratio, making it particularly susceptible to energy loss and dehydration [52,53]. This physiological constraint likely restricts long-distance movements and colonization of new areas, even when they appear climatically suitable. The energetic costs of maintaining body temperature and water balance may be particularly challenging during dispersal attempts, especially across suboptimal habitats or geographical barriers.

Behavioral and social factors further compound these limitations. The species exhibits strong site fidelity, with males typically dispersing only short distances from their natal roosts [50]. This limited dispersal behavior, combined with the species’ social structure and specific roost requirements, creates additional barriers to colonization. The need for suitable bamboo internodes, which must meet specific size and structural requirements [24], further restricts the species’ ability to establish new populations, even in areas with apparently suitable climate conditions.

### 4.4. Future Distribution Under Climate Change and Conservation Implications

Global warming is expected to have profound direct and indirect effects on species’ geographical distributions and suitable habitat extent [54]. Our projections reveal significant potential changes in *T. pachypus*’s distribution under different climate scenarios, with the SSP585 scenario showing the most dramatic habitat reduction. The total suitable habitat is projected to decrease by 36.1% by the 2050s and 64.4% by the 2090s under this scenario. Even under the more optimistic SSP126 scenario, substantial habitat reduction is expected, highlighting the species’ vulnerability to climate change regardless of emission pathways.

The projected northward and upward shift in *T. pachypus*’s distribution aligns with the anticipated movement of tropical/subtropical monsoon climate boundaries under global warming. While new potentially suitable areas may emerge in northern regions (reaching Hubei, Jiangxi, Zhejiang, and Anhui provinces in China), the southern parts of the current range, particularly in Southeast Asia, are likely to become less suitable. This asymmetric range shift presents complex challenges for conservation, as the species’ limited dispersal ability [50] may prevent it from tracking suitable climate conditions. Moreover, the Qinghai–Tibet Plateau acts as a significant barrier to further northward expansion in western regions [55], potentially creating ‘climate traps’ for some populations.

However, climate change impacts must be considered alongside other anthropogenic pressures. Ongoing urbanization and rural development continue to reduce bamboo forest coverage around human settlements. The combined effects of habitat fragmentation and climate change may create synergistic pressures on *T. pachypus* populations, potentially exceeding their adaptive capacity. Competition with sympatric species like *Tylonycteris tonkinensis*, which often uses the same bamboo tubes [56], may intensify as suitable habitat becomes more limited.

Based on these projections, we recommend a multi-faceted conservation strategy for *T. pachypus*. Priority should be given to protecting and maintaining bamboo forest corridors in areas predicted to remain suitable under future climate scenarios, particularly in South China and Southeast Asian countries. Conservation efforts should focus on the following: (1) preserving current core habitats that show resilience to climate change; (2) protecting potential climate refuge areas, especially in regions where topographic complexity may buffer climate impacts; (3) maintaining and restoring habitat connectivity to facilitate potential range shifts; and (4) implementing long-term monitoring programs to track population responses to climate change. These measures are crucial for ensuring the long-term survival of this unique bat species in the face of ongoing environmental changes.

## 5. Conclusions

Our study provides comprehensive insights into the current and future distribution of *Tylonycteris pachypus* under climate change scenarios through ensemble modeling approaches. The optimized ensemble model demonstrated excellent predictive performance (AUC: 0.981, TSS: 0.877) and identified three critical environmental variables—minimum temperature of the coldest month (40.90%), maximum temperature of the warmest month (38.38%), and precipitation of the wettest quarter (11.09%)—which collectively determine the species’ distribution patterns.

Our projections reveal concerning trends in habitat suitability, with the most severe reductions projected under the SSP585 scenario (64.4% decrease by 2090s). The asymmetric range shifts, characterized by potential northward expansion but significant southern range contraction, present complex conservation challenges given the species’ limited dispersal ability and specific habitat requirements. The interactive effects of climate change with habitat fragmentation and geographical barriers further complicate the species’ future persistence.

These findings emphasize the urgent need for targeted conservation strategies, particularly in areas predicted to maintain climatic suitability under future scenarios. While our model has limitations, notably the exclusion of interspecific competition and fine-scale habitat characteristics, it provides a robust framework for conservation planning. Future research should integrate these additional variables, along with detailed population dynamics and genetic connectivity analyses, to enhance our understanding of *T. pachypus*’s response to environmental changes.

This study advances our knowledge of how climate change may impact specialized species with restricted dispersal abilities and specific habitat requirements. The results underscore the importance of proactive conservation measures, including the preservation of bamboo forest corridors and potential climate refugia, to ensure the long-term survival of this unique bat species in an increasingly changing world.

## Figures and Tables

**Table 1:** Environmental variables used in this study.

Category	Environmental Variable	Unit	Participating in Modeling	% Contribution
Bioclimatic	Annual mean air temperature(Bio1)	°C		
	Mean diurnal range (Mean of monthly (max temp–min temp)) (Bio2)	°C	√	1.18
	Isothermality (Bio2/Bio7) (×100) (Bio3)	/		
	Variation in temperature seasonality (Bio4)	%		
	Max temperature of warmest month (Bio5)	°C	√	38.38
	Min temperature of coldest month (Bio6)	°C	√	40.90
	Temperature annual range (BIO5–BIO6) (Bio7)	°C		
	Mean temperature of wettest quarter (Bio8)	°C		
	Mean temperature of driest quarter (Bio9)	°C		
	Mean temperature of warmest quarter (Bio10)	°C		
	Mean temperature of coldest quarter (Bio11)	°C		
	Annual precipitation (Bio12)	mm		
	Precipitation of wettest month (Bio13)	mm		
	Precipitation of driest month (Bio14)	mm		
	Precipitation seasonality (Coefficient of variation) (Bio15)	/		
	Precipitation of wettest quarter (Bio16)	mm	√	11.09
	Precipitation of driest quarter (Bio17)	mm	√	6.32
	Precipitation of warmest quarter (Bio18)	mm	√	1.86
	Precipitation of coldest quarter (Bio19)	mm		
Topographic	Elevation (Alt)	M		
Anthropogenic	The land use and cover change (Lucc)	/	√	0.27

**Table 2:** Prediction accuracy of individual and ensemble models.

Model	AUC			TSS		
	Mean	SD	CV	Mean	SD	CV
GBM	0.963	0.015	0.016	0.766	0.083	0.108
MARS	0.947	0.022	0.023	0.773	0.083	0.108
XGBOOST	0.948	0.022	0.023	0.683	0.060	0.088
GLM	0.931	0.023	0.024	0.715	0.063	0.087
FDA	0.901	0.030	0.033	0.643	0.094	0.146
MAXENT	0.887	0.033	0.037	0.715	0.064	0.089
CTA	0.876	0.048	0.055	0.719	0.079	0.110
SRE	0.851	0.064	0.075	0.701	0.128	0.182
ANN	0.839	0.044	0.052	0.613	0.097	0.159
RF	0.956	0.013	0.013	0.646	0.064	0.100
Ensemble models	0.981	0.001	0.001	0.877	0.022	0.026

**Table 3:** Potential habitat area (×10^4^ km^2^) of *Tylonycteris pachypus* under current and future climate change scenarios.

Climate Scenarios	Poorly Suitable Habitat	Moderately Suitable Habitat	Highly Suitable Habitat	Total Area	Total Change	Area Percentage (%)
Current	83.244	71.368	291.893	446.505	/	37.10
SSP126_2050	75.209	53.864	187.914	316.988	−129.518	26.34
SSP126_2090	89.067	59.679	222.293	371.038	−75.467	30.83
SSP245_2050	75.837	55.819	192.804	324.460	−122.045	26.96
SSP245_2090	76.469	51.947	146.565	274.980	−171.525	22.85
SSP370_2050	67.626	52.974	171.727	292.327	−154.179	24.29
SSP370_2090	52.754	29.580	90.942	173.277	−273.228	14.40
SSP585_2050	75.859	53.244	156.306	285.409	−161.096	23.71
SSP585_2090	46.386	27.115	85.595	159.095	−287.410	13.22

## Data Availability

The data that support the findings of this study are available from the first author [K.C.] and correspondence author [L.W.] upon reasonable request.

## Supplementary Materials

The following supporting information can be downloaded at: https://www.mdpi.com/article/10.3390/ani15081164/s1, Figure S1 Heat map for environmental variables in Pearson correlation test.
