# Supplementary figures and images for: Biomod2 Modeling for Predicting Suitable Distribution of Bamboo Bat (Tylonycteris pachypus) Under Climate Change
Source: Animals (Basel). 2025 Apr 17;15(8):1164. doi: 10.3390/ani15081164 (PMC12023963; doi:10.3390/ani15081164)

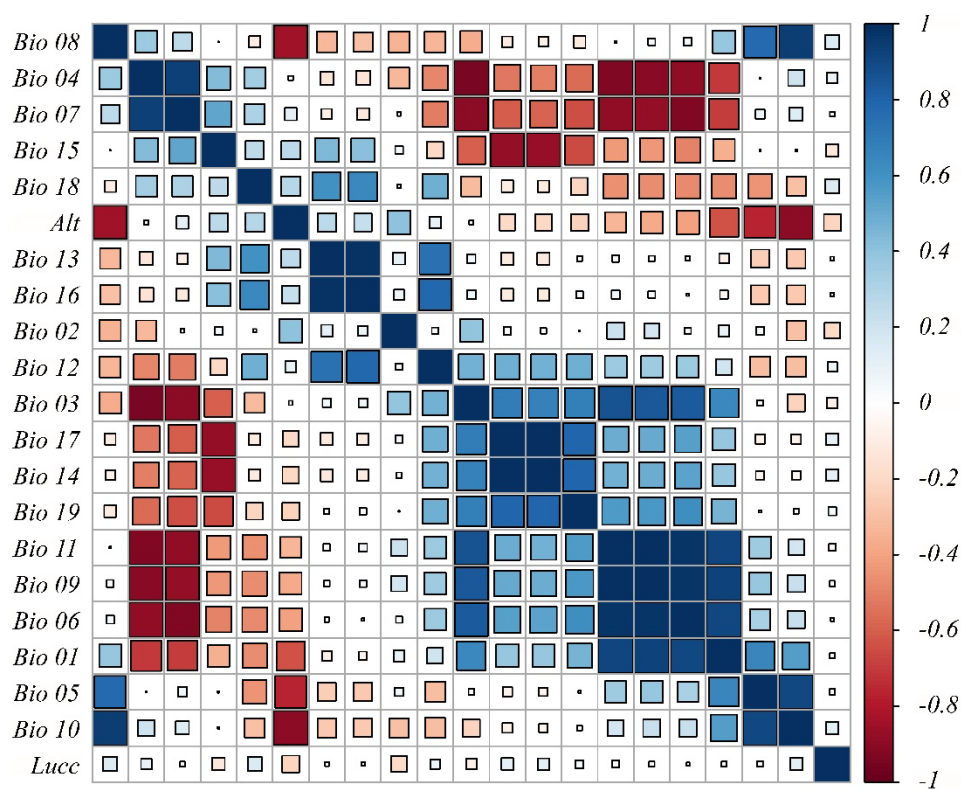

Supplementary Figure S1 Heat map for environmental variables in Pearson correlation test.

Supplement: Supplementary file 1 [file animals-15-01164-s001.zip › animals-3503520-supplementary.pdf]
